# Tumor engraftment in patient-derived xenografts of pancreatic ductal adenocarcinoma is associated with adverse clinicopathological features and poor survival

**DOI:** 10.1371/journal.pone.0182855

**Published:** 2017-08-30

**Authors:** Ilaria Pergolini, Vicente Morales-Oyarvide, Mari Mino-Kenudson, Kim C. Honselmann, Matthew W. Rosenbaum, Sabikun Nahar, Marina Kem, Cristina R. Ferrone, Keith D. Lillemoe, Nabeel Bardeesy, David P. Ryan, Sarah P. Thayer, Andrew L. Warshaw, Carlos Fernández-del Castillo, Andrew S. Liss

**Affiliations:** 1 Department of Surgery and the Andrew L. Warshaw, MD Institute for Pancreatic Cancer Research, Massachusetts General Hospital and Harvard Medical School, Boston, Massachusetts, United States of America; 2 Department of Surgery, Universita’ Politecnica delle Marche, Ancona, Italy; 3 Department of Pathology, Massachusetts General Hospital and Harvard Medical School, Boston, Massachusetts, United States of America; 4 Massachusetts General Hospital Cancer Center, Harvard Medical School, Boston, Massachusetts, United States of America; Vrije Universiteit Brussel, BELGIUM

## Abstract

Patient-derived xenograft (PDX) tumors are powerful tools to study cancer biology. However, the ability of PDX tumors to model the biological and histological diversity of pancreatic ductal adenocarcinoma (PDAC) is not well known. In this study, we subcutaneously implanted 133 primary and metastatic PDAC tumors into immunodeficient mice. Fifty-seven tumors were successfully engrafted and even after extensive passaging, the histology of poorly-, moderately-, and well-differentiated tumors was maintained in the PDX models. Moreover, the fibroblast and collagen contents in the stroma of patient tumors were recapitulated in the corresponding PDX models. Analysis of the clinicopathological features of patients revealed xenograft tumor engraftment was associated with lymphovascular invasion (*P* = 0.001) and worse recurrence-free (median, 7 vs. 16 months, log-rank *P* = 0.047) and overall survival (median, 13 vs. 21 months, log-rank *P* = 0.038). Among successful engraftments, median time of growth required for reimplantation into new mice was 151 days. Reflective of the inherent biological diversity between PDX tumors with rapid (<151 days) and slow growth, differences in their growth were maintained during extensive passaging. Rapid growth was additionally associated with lymph node metastasis (*P* = 0.022). The association of lymphovascular invasion and lymph node metastasis with PDX formation and rapid growth may reflect an underlying biological mechanism that allows these tumors to adapt and grow in a new environment. While the ability of PDX tumors to mimic the cellular and non-cellular features of the parental tumor stroma provides a valuable model to study the interaction of PDAC cells with the tumor microenvironment, the association of successful engraftment with adverse clinicopathological features suggests PDX models over represent more aggressive forms of this disease.

## Introduction

Pancreatic ductal adenocarcinoma (PDAC) is the most common pancreatic cancer, with more than 53,000 cases diagnosed per year in the United States. Only 8% of these patients survive beyond five years, making PDAC the fourth leading cause of cancer-related deaths [1]. The ineffectiveness of treatments and the scant improvement of survival outcomes may be ascribed to the fact that PDAC has historically been modeled as a single disease entity. By contrast, recent advances in genomics have revealed the heterogeneity of this disease [2–4]. Although activating mutations in *KRAS* occur in ~90% of PDAC, there are few additional genes (such as *TP53* and *SMAD4*) commonly mutated or inactivated in pancreatic cancer [5]. Contributing to the challenges associated with treating PDAC is a highly desmoplastic stroma that promotes the aggressive local growth of the tumor and the intrinsic chemo-resistance of the cancer cells [6–9]. Thus, modeling the tumor microenvironment and its crosstalk with the cancer cells is particularly important in developing new therapies for PDAC.

Several *in vitro* and *in vivo* preclinical models are available to study the biology of cancer, including cell lines and xenograft tumors derived from them, genetically-engineered mouse models, organoids, and patient-derived xenograft (PDX) tumor models [10,11]. Among these, PDX tumors have the advantage of mimicking the genetic complexity of human PDAC in a platform that has the potential to recapitulate many of the features of the tumor microenvironment. Moreover, a model that faithfully reflects the original tumor biology may predict clinical outcomes and allow for the development of personalized targeted therapies [12–15]. However, the growth of PDX tumors from pancreatic cancer is variable and the determinants of their growth are unknown [16–19].

Understanding the dynamic behind xenograft tumor formation, the features that affect the success of tumor engraftment, and the prognostic implications could be an important key for new perspectives in the knowledge of pancreatic cancer. The aims of our study were to identify clinical and pathological factors associated with successful tumor engraftment and xenograft growth rate, and to evaluate whether tumor engraftment and xenograft rate of growth were prognostic of patient outcomes. We also analyzed PDX tumors to assess the ability of these models to reproduce the histological features of the original pancreatic tumors.

## Materials and methods

### Patient population and tumor samples

All study participants provided IRB-approved informed consent for their medical records and tissue samples to be used in this study. Patient clinical data was entered into a de-identified clinical database allowing for the anonymous analysis of demographic, clinical and pathological variables. We collected fresh tumor samples from 133 patients with histologically-confirmed stages I-IV PDAC who underwent surgery for curative intent, diagnostic laparoscopy, or palliation of symptoms at the Massachusetts General Hospital between April, 2009 and July, 2012.

### Xenograft tumors

Patient tumor samples were mechanically minced into small fragments (1–2 mm^3^) and either implanted into mice or cryopreserved in freezing media (10% DMSO, 20% FBS, 70% DMEM/Ham’s F-12 50/50 supplemented with 1% PS) for future implantation. Cryopreserved tumors were rapidly thawed in a 37°C water bath and washed twice with PBS prior to implantation. For tumor implantation, 6–8 week-old nu/j mice (Jackson Laboratory) were anesthetized with Isoflurane and a small incision made on the dorsal flank. Approximately 70–100 mg of tumor tissue coated in 50–100 of μl of Matrigel (Corning, 354248) was subcutaneously implanted into the flank of each mouse and the incision was closed with a single suture (4–0 Coated Vicryl, Ethicon). Mice were administered buprenorphine as needed as an analgesic. Patient tumors were implanted in a median number of 4 mice (range 1–5). In 10.5% of cases the tumor tissue was enough for only one mouse. Mice were monitored weekly for tumor growth. Mice that lacked a palpable tumor after 6 months were removed from the study and the patient tumors were categorized as no engraftment. Successful tumor engraftment was defined as tumors that grew large enough (1 cm) to be reimplanted into new mice. Mice with tumors < 1 cm after 180 days were retained in the study until tumors reached sufficient size for reimplantation. Tumors implanted in mice that were removed from the study due to health reasons before the tumor reached an appropriate size for reimplantation were categorized as no engraftment. For tumors that successfully formed xenografts, we recorded the time of growth between initial implantation and reimplantation into new mice. The presence of pancreatic adenocarcinoma in xenograft tumors was confirmed by histological analysis of hematoxylin and eosin (H&E) stained sections by pathologists with a special interest in pancreatic cancer (M.M.K. and M.W.R). Maximum tumor size in this study did not exceed 1.5 cm. Mice were euthanized by CO_2_ asphyxiation in accordance to the guidelines set forth in the American Veterinary Medical Association (AVMA) Guidelines for the Euthanasia of Animals.

### Immunohistochemistry

Representative sections of primary human tumors were stained with a 1:50 dilution of a goat polyclonal antibody specific to SMAD4 (sc-1909, Santa Cruz Biotechnology, Dallas, TX, USA) using the Bond RX IHC staining platform (Leica Biosystems, Buffalo Grove, IL, USA) with BOND Epitope Retrieval Solution 2 (Leica Biosystems, AR9640). The staining of SMAD4 was scored by a pathologist and SMAD4 expression was considered preserved (positive) when detected in the nucleus and/or cytoplasm of PDAC cells. Staining of stromal cells in each tumor section was used as an internal positive control. For analysis of cancer-associated fibroblasts, deparaffinized sections of patient and PDX tumors were stained with a 1:200 dilution of a rabbit polyclonal antibody specific to alpha smooth muscle actin (ab5694, Abcam, Cambridge, MA, USA) as described previously [20]. The collagen content of tumors was visualized by picrosirius red staining (Picro Sirius Red Stain Kit, Abcam, Cambridge, MA, USA).

### Genetic analysis of patient tumors

DNA extracted from formalin-fixed paraffin-embedded tumor samples from 20 patients was analyzed for somatic mutations using SNaPshot multiplex assays (Applied Biosystems) [21]. Common to these assays were the tests for variants in loci found in *APC*, *BRAF*, *CTNNB1*, *EGFR*, *KIT*, *KRAS*, *NOTCH1*, *NRAS*, *PI3KA*, *PTEN*, and *TP53*. On average, approximately 5% mutant allele is sufficient for detection in these assays. Only mutations in *KRAS* and *TP53* were detected in our series.

### Outcome measures

The primary outcome measures of this study were successful tumor engraftment and xenograft rate of growth. We also evaluated whether tumor engraftment and xenograft growth rate were associated with patient recurrence-free survival (RFS) and overall survival (OS). RFS was defined as time between surgery and evidence of disease recurrence or death from any cause; analyses of RFS were restricted to patients with resectable primary tumors and no evidence of metastatic disease (n = 112). OS was defined as time between surgery and death from any cause; analyses of OS were performed in the entire study population. Follow-up continued through February, 2017.

### Statistical analysis

We evaluated the associations of clinical and pathological features with tumor engraftment and xenograft rate of growth using univariate analyses. Analyses of categorical data were performed using chi-square or Fisher exact tests, where appropriate; continuous data were analyzed with Mann-Whitney U test. Associations of tumor engraftment and xenograft growth rate with patient survival were analyzed using log-rank tests and multivariable-adjusted Cox proportional hazards regression adjusting for potential confounders. Kaplan-Meier survival curves, median survival time, and two- and five-year survival rates were also presented. Cox regression models adjusted for patient age and sex, receipt of neoadjuvant and adjuvant therapy, American Joint Committee on Cancer (7^th^ edition) clinical stage, tumor differentiation grade, presence of lymphovascular invasion, surgical margins, and tumor location. Statistical significance was set at *P*<0.05 and all hypothesis tests were two-sided. Statistical analyses were conducted using SAS software (version 9.4; SAS Institute, Cary, NC).

## Results

Primary or metastatic tumor samples were collected from 133 patients with histologically-confirmed diagnosis of PDAC. The baseline characteristics of patients in our study are presented in Tables 1 and 2. The median age of the patients in our study was 68 (range 35–93). Fifty-seven (43%) tumors implanted into immunodeficient mice were successfully engrafted, while 76 (57%) failed to do so. A number of studies have reported different methods of cryopreserving tumors for future growth as xenograft tumors [22,23]. In our study, there was no significant difference in the engraftment rate between freshly implanted (n = 24) and cryopreserved (n = 109) tumors (50% vs 41%, *P* = 0.498).

**Table 1:** Baseline demographic and clinical characteristics of 133 patients with resected pancreatic ductal adenocarcinoma by tumor engraftment status.

	Overall	Tumor engraftment		
		Yes	No	*P* value
No. Patients	133	57 (43%)	76 (57%)	
Men, n (%)	70 (53%)	34 (60%)	36 (47%)	0.160
Age, median (IQR)	68 (18)	66 (16)	70 (18.5)	0.730
Serum CA19-9, median (IQR)	128 (376.6)	118 (347.0)	128.5 (436.5)	0.916
Body mass index, median (IQR)	25.9 (6.0)	26.8 (6.3)	25.2 (6.0)	0.390
Diabetes, n (%)				
*No*	101 (76%)	41 (72%)	60 (79%)	0.345
*Yes*	32 (24%)	16 (28%)	16 (21%)	
New-onset or worsening diabetes, n (%)*				
*No*	11 (34%)	6 (37%)	5 (31%)	0.809
*Yes*	20 (63%)	10 (63%)	10 (63%)	
*Unknown*	1 (3%)	-	1 (6%)	
Neoadjuvant therapy, n (%)				
*No*	91 (68%)	42 (74%)	49 (64%)	0.258
*Yes*	42 (32%)	15 (26%)	27 (36%)	
Resection, n (%)				
*No*	17 (13%)	9 (16%)	8 (11%)	0.368
*Yes*	116 (87%)	48 (84%)	68 (89%)	
Metastatic disease, n (%)				
*No*	112 (84%)	45 (79%)	67 (88%)	0.149
*Yes*	21 (16%)	12 (21%)	9 (12%)	
Type of resection, n (%)				
*Whipple*	91 (68%)	34 (59%)	57 (75%)	0.131
*Middle*	4 (3%)	1 (2%)	3 (4%)	
*Distal*	20 (15%)	13 (23%)	7 (9%)	
*Total*	1 (1%)	-	1 (1%)	
*No resection*	17 (13%)	9 (16%)	8 (11%)	
Adjuvant therapy, n (%)**				
*No*	30 (27%)	10 (22%)	20 (30%)	0.421
*Yes*	79 (70%)	33 (73%)	46 (69%)	
*Unknown*	3 (3%)	2 (5%)	1 (1%)	
Recurrence, n (%)**				
*No*	28 (25%)	9 (20%)	19 (28%)	0.317
*Yes*	84 (75%)	36 (80%)	48 (72%)	
Site of recurrence, n (%)***				
*Locoregional*	21 (25%)	9 (25%)	12 (25%)	0.488
*Distant*	54 (64%)	24 (67%)	30 (63%)	
*Both locoregional and distant*	8 (10%)	2 (5%)	6 (12%)	
*Unknown*	1 (1%)	1 (3%)	-	

Abbreviations: IQR, interquartile range.

*Among patients with diabetes mellitus (n = 32).

** Among patients undergoing resection with curative intent and absence of metastatic disease (n = 112).

*** Among patients with evidence of recurrence following resection with curative intent (n = 84).

**Table 2:** Pathological characteristics of 133 patients with resected pancreatic ductal adenocarcinoma by tumor engraftment status.

	Overall	Tumor engraftment		
		Yes	No	*P* value
No. Patients	133	57 (43%)	76 (57%)	
Tumor differentiation grade, n (%)				
*Well differentiated*	7 (5%)	3 (5%)	4 (5%)	0.846
*Moderately differentiated*	65 (49%)	26 (46%)	39 (52%)	
*Poorly differentiated*	53 (40%)	24 (42%)	29 (38%)	
*Unknown*	8 (6%)	4 (7%)	4 (5%)	
AJCC 7th ed. stage, n (%)				
*IA*	1 (1%)	1 (2%)	-	0.333
*IB*	6 (4%)	2 (3%)	4 (5%)	
*IIA*	17 (13%)	5 (9%)	12 (16%)	
*IIB*	88 (66%)	37 (65%)	51 (67%)	
*III*	-	-	-	
*IV*	21 (16%)	12 (21%)	9 (12%)	
AJCC 7th ed. pT, n (%)*				
*pT1*	2 (2%)	1 (2%)	1 (2%)	0.928
*pT2*	11 (10%)	4 (9%)	7 (10%)	
*pT3*	99 (88%)	40 (89%)	59 (88%)	
*pT4*	-	-	-	
AJCC 7th ed. pN, n (%)*				
*pN0*	24 (21%)	8 (18%)	16 (24%)	0.440
*pN1*	88 (79%)	37 (82%)	51 (76%)	
Tumor location, n (%)				
*Body/Tail*	30 (23%)	18 (32%)	12 (16%)	0.031
*Head/Uncinante*	103 (77%)	39 (68%)	64 (84%)	
Size cm, median (IQR)*	3.0 (1.5)	3.5 (1.7)	2.8 (1.6)	0.051
Lymphovascular invasion, n (%)				
*Absent*	40 (30%)	8 (14%)	32 (42%)	0.001
*Present*	81 (61%)	43 (75%)	38 (50%)	
*Unknown*	12 (9%)	6 (11%)	6 (8%)	
Perineural invasion, n (%)				
*Absent*	6 (4%)	2 (3%)	4 (5%)	0.681
*Present*	110 (83%)	46 (81%)	64 (84%)	
*Unknown*	17 (13%)	9 (16%)	8 (11%)	
Surgical margins, n (%)*				
*R0*	98 (87%)	41 (91%)	57 (85%)	0.532
*R1*	13 (12%)	4 (9%)	9 (13%)	
*R2*	1 (1%)	-	1 (2%)	
SMAD4, n (%)**				
*Retained*	40 (62%)	20 (69%)	20 (57%)	0.331
*Lost*	24 (38%)	9 (31%)	15 (43%)	

Abbreviations: IQR, interquartile range.

*Among patients undergoing resection with curative intent and absence of metastatic disease (n = 112).

**Among tumors with available SMAD4 immunohistochemistry (n = 64).

**Fig 1:**
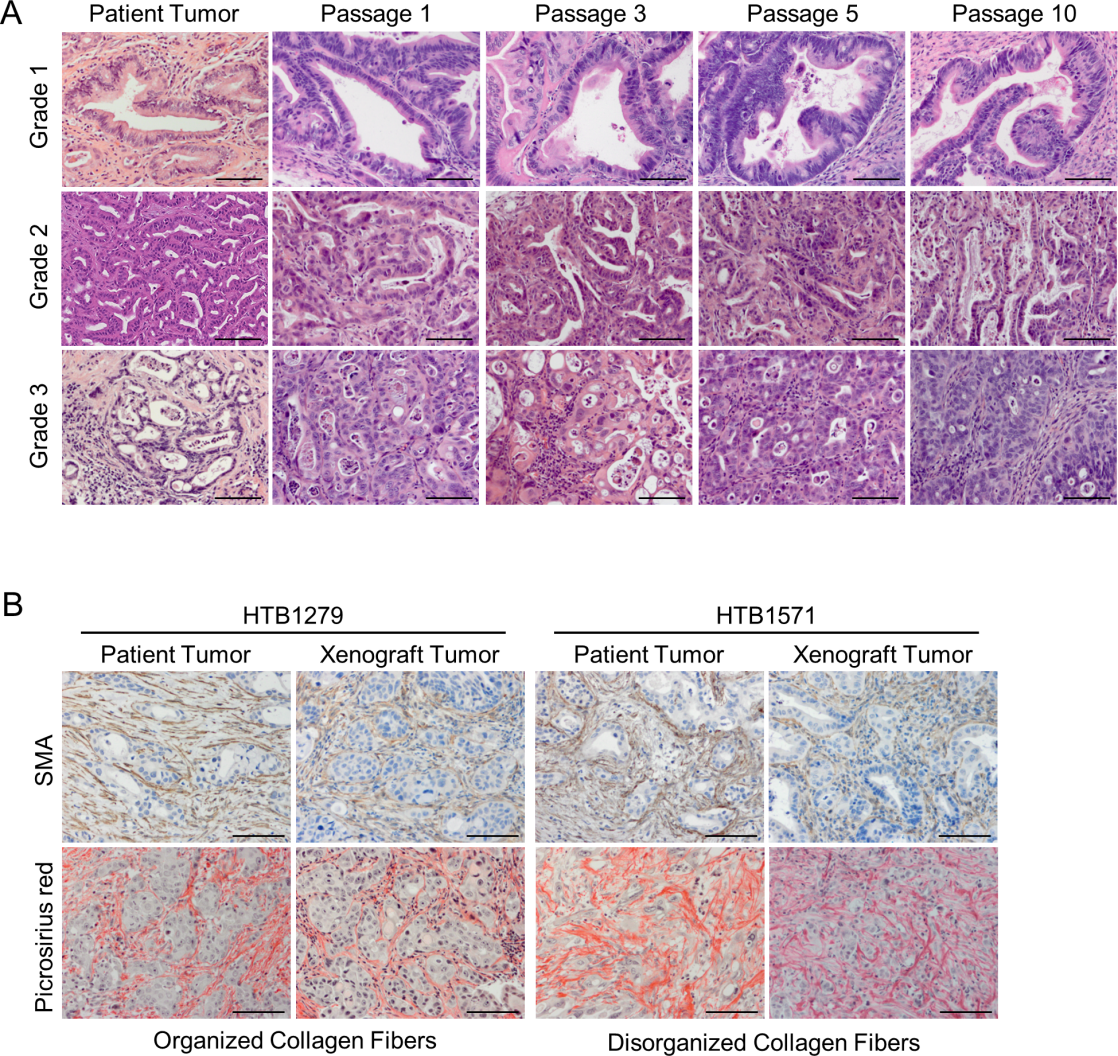
PDX models of PDAC retain the histological and stromal features of the parental tumor. (A) H&E staining of well-differentiated (grade 1), moderately-differentiated (grade 2) and poorly-differentiated (grade 3) tumors. The primary patient tumor and corresponding passages of the PDX models are shown. Scale bars = 100 μm. (B) Immunohistochemistry for α-smooth muscle actin (SMA; top panels) and picrosirius red staining for collagen (bottom panels) was performed on representative PDX models and corresponding patient tumors. Scale bars = 100 μm.

Histological analysis of the xenograft tumors revealed that the grade of differentiation of the human tumors was retained in each of the corresponding xenografts. Moreover, the histology of poorly-, moderately-, and well-differentiated tumors was retained through at least 10 generations in mice (Fig 1A). Characteristic of the stroma of PDAC tumors is the presence of cancer associated fibroblasts and abundance of collagen. The corresponding xenograft tumors contain α-smooth muscle actin-expressing fibroblasts similarly to patient tumors (Fig 1B). Furthermore, picrosirius red staining revealed that collagen content of xenograft tumors was largely composed of either organized or disorganized fibers and these collagen subtypes were similarly found within the malignant epithelium of the corresponding patient tumor (Fig 1B). Collectively, these results demonstrate that patient-derived xenograft models of PDAC retain the histological and microenvironmental characteristics of the original tumor.

### Clinical and genetic characteristics, pathological features, and tumor engraftment status

To gain insight into the factors that determine tumor engraftment, we evaluated the demographic, clinical, and pathological characteristics of patients based on tumor engraftment status (Tables 1 and 2). We found no significant associations between demographic or clinical features and tumor engraftment. Neoadjuvant chemotherapy has become increasingly common in patients with PDAC and is associated with increased fibrosis and a reduction in viable cancer cells, which could influence the ability of a tumor to successfully establish a xenograft [24]. Notably, 42 (32%) patients in our series received neoadjuvant chemotherapy and this did not adversely affect tumor engraftment (Table 1).

Analysis of pathological features of patient tumors demonstrated that lymphovascular invasion and location of the primary tumor within the pancreas were significantly associated with tumor engraftment. Tumors with successful engraftment had higher frequency of lymphovascular invasion (43/57, 75%) compared to tumors that did not form xenografts (38/76, 50%; *P* = 0.001). Among patients whose tumors successfully engrafted, primary tumors were located in the body and tail of the pancreas in 32% (18/57) of cases; in contrast, only 16% (12/76) of primary tumors that did not engraft were located in the pancreatic body and tail (*P* = 0.031). Features such as tumor grade, nodal metastases, or type of implanted tumor tissue (i.e., primary vs. metastatic) were not significantly associated with engraftment status.

**Fig 2:**
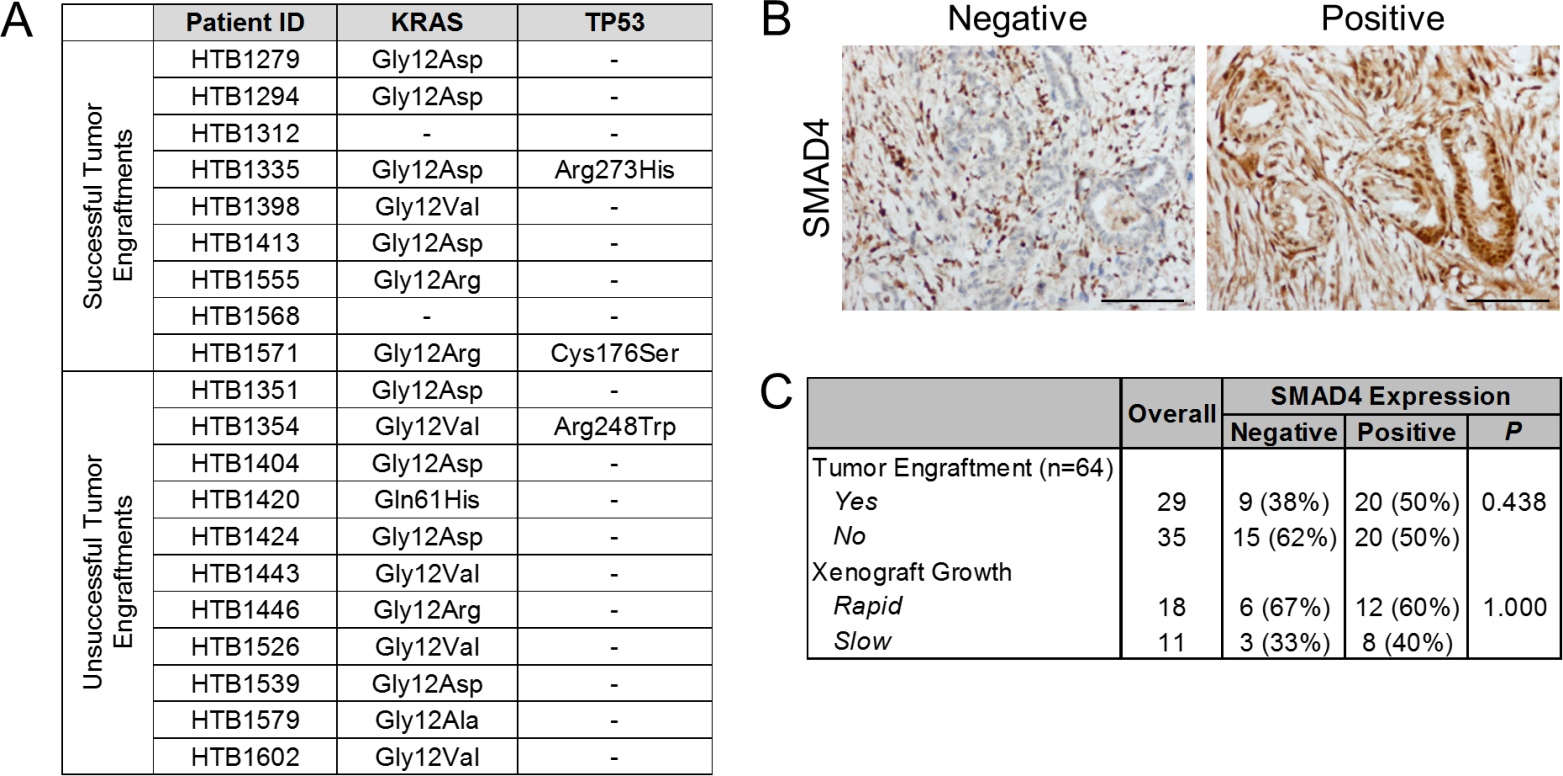
Molecular characteristics of patient tumors do not predict tumor engraftment. (A) Genetic analysis of *KRAS* and *TP53* in patient tumors. Amino acid changes resulting from mutations are listed. Samples for which mutations were not detected are indicated by a dash. (B) Representative positive and negative immunohistochemical staining of SMAD4 in primary patient tumors. Staining of stromal cells on each slide served as a positive control. Scale bars = 100 μm. (C) Summary of SMAD4 expression in patient tumors that were successfully or unsuccessfully engrafted in mice.

To evaluate whether common molecular alterations found in PDAC are associated with tumor engraftment, we performed genetic analysis of *KRAS* and *TP53*, and immunohistochemistry for SMAD4. Genetic analysis of engrafted and non-engrafted tumors revealed 18/20 (90%) tumors contained activating mutations in codon 12 or 61 of *KRAS*, while hot spot mutations in *TP53* were identified in three (15%) tumors (Fig 2A). However, there was no association between these mutations and the success of tumor engraftment. SMAD4 is frequently inactivated in PDAC through both genetic and epigenetic mechanisms [25]. Therefore, we employed immunohistochemistry to evaluate the expression of SMAD4 in 64 patient tumors, 45% of which were successfully engrafted (Fig 2B). The expression of SMAD4 was lost in 38% of tumors, and there was no significant difference in the loss of SMAD4 expression between engrafted and non-engrafted tumors (31% vs 43%, *P* = 0.331, Fig 2C). Taken together, these results suggest that alterations in these core PDAC pathways are not predictive of tumor engraftment.

### Clinical characteristics, pathological features, and xenograft growth rate

**Fig 3:**
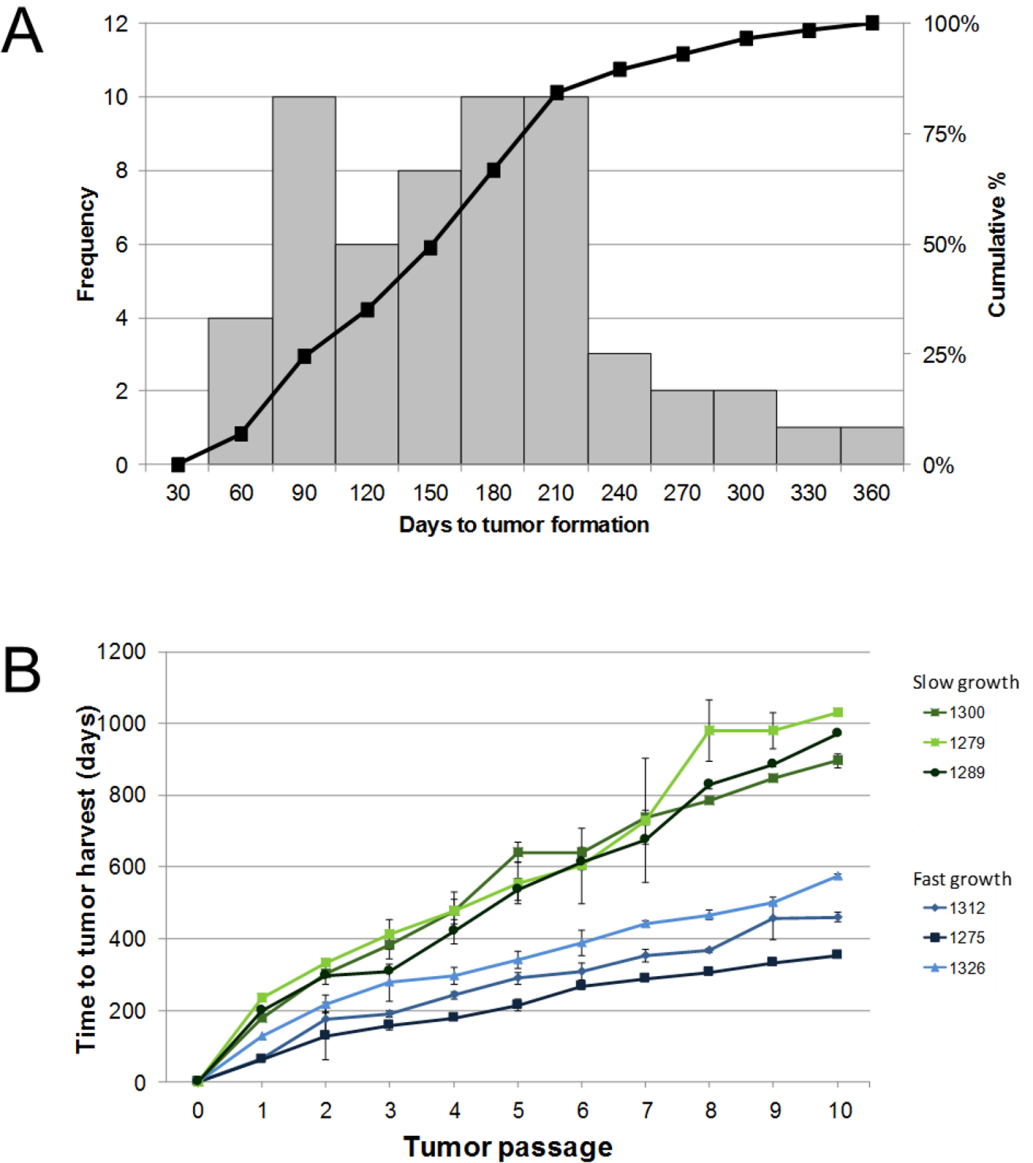
The time to tumor engraftment correlates with the rate of PDX tumor growth. (A) Time to tumor engraftment was grouped in 30-day intervals and the frequency of their occurrence is shown on the left y-axis. The cumulative percentage of tumors engrafted over time is shown on the right y-axis. (B) The growth of PDX tumors that exhibited rapid and slow engraftment. The median and standard deviation of tumors for passages 2–10 are shown. Passage 1 tumors represent the founding PDX tumor from which subsequent PDX passages were derived.

Among successful engraftments, the median time of growth required for reimplantation into new mice was 151 days (range 39–346 days; Fig 3A). Xenograft tumors that were reimplanted in less than 151 days were defined as having a rapid growth rate. Analysis of representative rapid and slow (≥ 151 days) xenograft tumor lines revealed that the relative differences in the growth rate of these tumors was maintained for at least 10 generations (Fig 3B). Since these differences in the time to tumor engraftment may be related to the inherent characteristics of the tumor biology, we performed a comparison of the clinical (Table 3) and pathological (Table 4) features between tumors with rapid and slow growth. Rapid growth was significantly associated with patients of male gender (*P* = 0.020), primary tumors located in the head of the pancreas (*P* = 0.029), and lymph node metastases (*P* = 0.022). Collectively, our results suggest that both tumor engraftment and xenograft growth rate are associated with adverse pathological features (e.g. lymphovascular for tumor engraftment and lymph node metastases for xenograft growth rate).

**Table 3:** Baseline demographic and clinical characteristics of 57 patients with patient-derived PDAC xenografts based on xenograft growth rate.

	Overall	Xenograft Growth		
		Rapid	Slow	*P* value
No. patients	57	28 (49%)	29 (51%)	
Men, n (%)	34 (60%)	21 (75%)	13 (45%)	0.020
Age, median (IQR)	66 (16.0)	66 (11.5)	71 (24)	0.193
Serum CA19-9, median (IQR)	118 (347.0)	145 (376.5)	94 (195.0)	0.429
Body mass index, median (IQR)	26.8 (6.3)	27.3 (7.9)	26.3 (5.3)	0.334
Diabetes, n (%)				
*No*	41 (72%)	20 (71%)	21 (72%)	0.934
*Yes*	16 (28%)	8 (29%)	8 (28%)	
New-onset or worsening diabetes, n (%)*				
*No*	6 (37%)	2 (25%)	4 (50%)	0.608
*Yes*	10 (63%)	6 (75%)	4 (50%)	
Neoadjuvant therapy, n (%)				
*No*	42 (74%)	20 (71%)	22 (76%)	0.704
*Yes*	15 (26%)	8 (29%)	7 (24%)	
Resection, n (%)				
*No*	9 (16%)	4 (14%)	5 (17%)	1.00
*Yes*	48 (84%)	24 (86%)	24 (83%)	
Metastatic disease, n (%)				
*No*	45 (79%)	23 (82%)	22 (76%)	0.561
*Yes*	12 (21%)	5 (18%)	7 (24%)	
Type of resection, n (%)				
*Whipple*	34 (59%)	20 (72%)	14 (48%)	0.253
*Middle*	1 (2%)	-	1 (4%)	
*Distal*	13 (23%)	4 (14%)	9 (31%)	
*Total*	-	-	-	
*No resection*	9 (16%)	4 (14%)	5 (17%)	
Adjuvant therapy, n (%)**				
*No*	10 (22%)	3 (13%)	7 (32%)	0.281
*Yes*	33 (73%)	18 (78%)	15 (68%)	
*Unknown*	2 (5%)	2 (9%)	-	
Recurrence, n (%)**				
*No*	9 (20%)	3 (13%)	6 (27%)	0.284
*Yes*	36 (80%)	20 (87%)	16 (73%)	
Site of recurrence, n (%)***				
*Locoregional*	9 (25%)	5 (25%)	4 (25%)	0.412
*Distant*	24 (67%)	13 (65%)	11 (69%)	
*Both locoregional and distant*	2 (5%)	2 (10%)	-	
*Unknown*	1 (3%)	-	1 (6%)	

Abbreviations: IQR, interquartile range.

*Among patients with diabetes mellitus (n = 16).

** Among patients undergoing resection with curative intent and absence of metastatic disease (n = 45).

*** Among patients with evidence of recurrence following resection with curative intent (n = 36).

**Table 4:** Baseline pathological characteristics of 57 patients with patient-derived PDAC xenografts based on xenograft growth rate.

	Overall	Xenograft Growth		
		Rapid	Slow	*P* value
No. patients	57	28 (49%)	29 (51%)	
Tumor differentiation grade, n (%)				
*Well differentiated*	3 (5%)	1 (4%)	2 (7%)	0.728
*Moderately differentiated*	26 (46%)	14 (50%)	12 (41%)	
*Poorly differentiated*	24 (42%)	11 (39%)	13 (45%)	
*Unknown*	4 (7%)	2 (7%)	2 (7%)	
AJCC 7th ed. stage, n (%)				
*IA*	1 (2%)	-	1 (3.5%)	0.106
*IB*	2 (3%)	1 (3%)	1 (3.5%)	
*IIA*	5 (9%)	-	5 (17%)	
*IIB*	37 (65%)	22 (79%)	15 (52%)	
*III*	-	-	-	
*IV*	12 (21%)	5 (18%)	7 (24%)	
AJCC 7th ed. pT, n (%)*				
*pT1*	1 (2%)	-	1 (4%)	0.304
*pT2*	4 (9%)	1 (4%)	3 (14%)	
*pT3*	40 (89%)	22 (96%)	18 (82%)	
*pT4*	-	-	-	
AJCC 7th ed. pN, n (%)*				
*pN0*	8 (18%)	1 (4%)	7 (32%)	0.022
*pN1*	37 (82%)	22 (96%)	15 (68%)	
Tumor location, n (%)				
*Body/Tail*	18 (32%)	5 (18%)	13 (45%)	0.029
*Head/Uncinante*	39 (68%)	23 (82%)	16 (55%)	
Size cm, median (IQR)*	3.5 (1.7)	3.3 (1.7)	3.7 (1.5)	0.910
Lymphovascular invasion, n (%)				
*Absent*	8 (14%)	2 (7%)	6 (21%)	0.140
*Present*	43 (75%)	24 (86%)	19 (65%)	
*Unknown*	6 (11%)	2 (7%)	4 (14%)	
Perineural invasion, n (%)				
*Absent*	2 (3%)	-	2 (7%)	0.489
*Present*	46 (81%)	24 (86%)	22 (76%)	
*Unknown*	9 (16%)	4 (14%)	5 (17%)	
Surgical margins, n (%)*				
*R0*	41 (91%)	22 (96%)	19 (86%)	0.346
*R1*	4 (9%)	1 (4%)	3 (14%)	
*R2*	-	-	-	
SMAD4, n (%)**				
*Retained*	20 (69%)	12 (67%)	8 (73%)	1.000
*Lost*	9 (31%)	6 (33%)	3 (27%)	

Abbreviations: IQR, interquartile range.

*Among patients undergoing resection with curative intent and absence of metastatic disease (n = 45).

**Among tumors with available SMAD4 immunohistochemistry (n = 29).

### Tumor engraftment, rate of growth, and survival outcomes

To determine the prognostic value of tumor engraftment, we analyzed the relationship between patient survival outcomes and PDX formation and growth rate. Overall, 30 (22.6%) patients of our cohort were alive at the end of follow-up period, and among them the median follow up time was 45 months. In patients who underwent resection with curative intent without evidence of metastatic disease, the median, 2-year, and 5-year RFS was 10 months (95% CI 7–15), 25.3%, and 12.7%, respectively. In the entire study population, the median, 2-year, and 5-year OS was 16 months (95% CI 13–19), 38.0%, and 14.7%, respectively.

**Fig 4:**
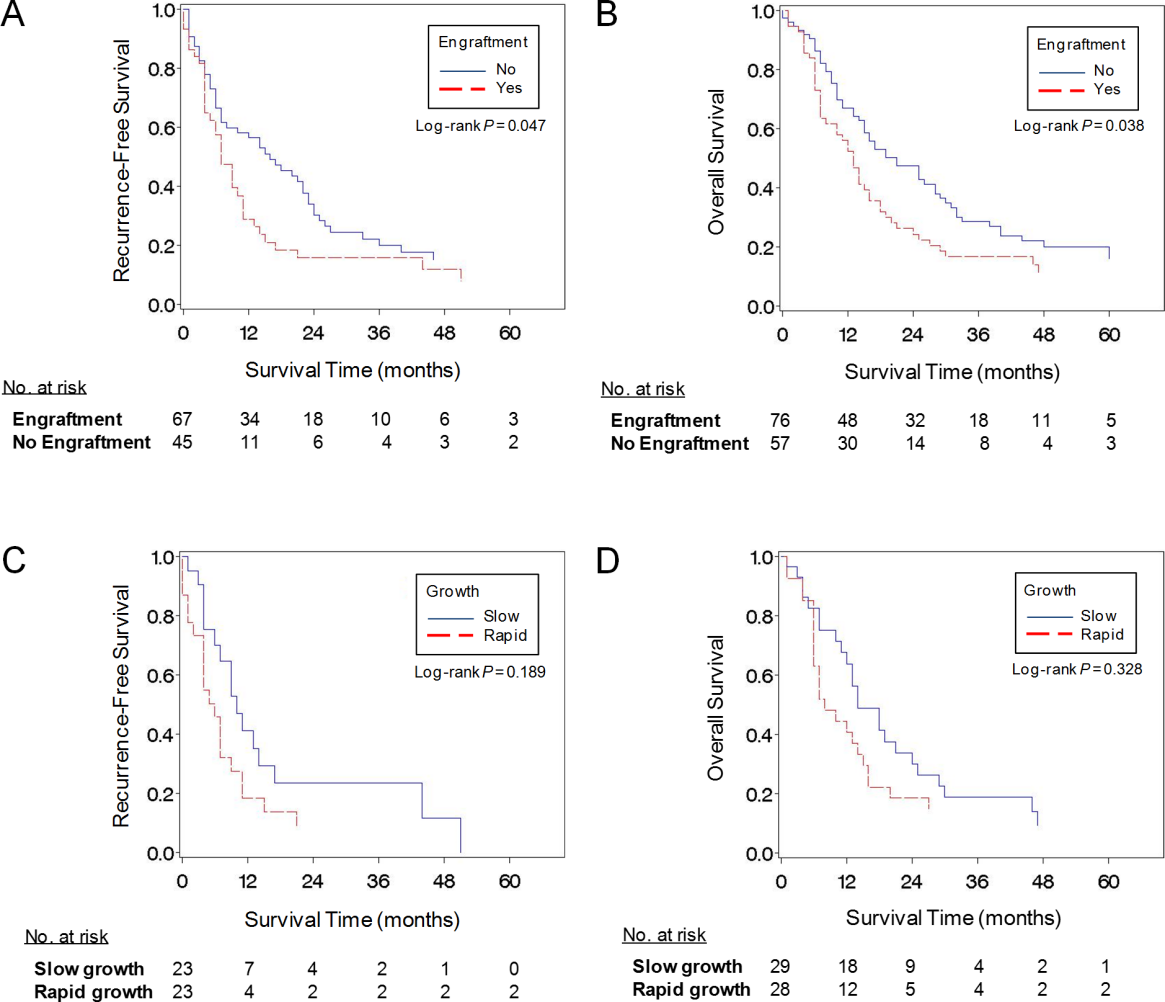
PDAC tumor engraftment in mice is associated with poor patient outcomes. (A) Kaplan-Meier curves of recurrence-free survival and tumor engraftment for patients with resectable primary tumors and no evidence of metastatic disease. (B) Kaplan-Meier curves of overall survival and tumor engraftment for the entire study population. (C) Kaplan-Meier curves of recurrence-free survival and rate of tumor engraftment for patients with resectable primary tumors and no evidence of metastatic disease. (D) Kaplan-Meier curves of overall survival and rate of tumor engraftment for the entire study population.

Patients with tumors that successfully engrafted had significantly shorter RFS (median, 7 vs. 16 months, log-rank *P =* 0.047; Fig 4A) and OS (median, 13 vs. 21 months, log-rank *P* = 0.038; Fig 4B) compared to patients with tumors that failed to engraft. Moreover, the multivariable-adjusted survival analyses revealed tumor engraftment as an independent predictor of worse RFS (HR 2.05, 95% CI 1.20–3.50, *P* = 0.009) (Table 5). Survival analyses based on xenograft growth rate did not show significant associations with patient survival outcomes, although analyses were limited by sample size (Table 5, Fig 4C and 4D). Patients with rapid-growing xenografts had shorter RFS (median, 6 vs. 10 months, log-rank *P* = 0.189) and OS (median, 8 vs. 14, log-rank *P* = 0.328) than those with slow-growing xenografts, although these differences were not statistically significant.

**Table 5:** Recurrence-free and overall survival by tumor engraftment and xenograft growht rate.

** **	**Recurrence-Free Survival**					
	**No. patients**	**Median (Months)**	**2-year**	**5-year**	HR* **(95% CI)**	***P***
**Tumor engraftment**						
No engraftment	67	16.0	30.3%	15.2%	1.00 (reference)	
Engraftment	45	7.0	16.9%	8.5%	2.05 (1.20–3.50)	0.009
**Xenograft growth rate**						
Slow growth	22	10.0	23.5%	0.0%	1.00 (reference)	
Rapid growth	23	6.0	10.5%	10.5%	2.37 (0.90–6.25)	0.082
** **	**Overall Survival**					
	**No. patients**	**Median (Months)**	**2-year**	**5-year**	HR* **(95% CI)**	***P***
**Tumor engraftment**						
No engraftment	76	21.0	48.7%	16.5%	1.00 (reference)	
Engraftment	57	13.0	24.3%	11.2%	1.44 (0.90–2.31)	0.125
**Xenograft growth rate**						
Slow growth	29	14.0	30.0%	9.4%	1.00 (reference)	
Rapid growth	28	8.0	18.5%	14.8%	1.63 (0.79–3.38)	0.190

Abbreviations: HR, hazard ratio.

*Cox proportional hazards regression adjusting for patient age, sex, neoadjuvant therapy, adjuvant therapy, American Joint Committee on Cancer stage, tumor location, tumor grade of differentiation, lymphovascular invasion, and resection margins.

## Discussion

The genetic heterogeneity of PDAC and the intense desmoplastic reaction of the tumor stroma have made improvements in survival outcomes difficult to achieve. Preclinical models that closely recapitulate the complexity of human pancreatic cancer are indispensable to study the biology of this disease and assess novel therapeutic agents. In this setting, PDX tumor models of PDAC allow for the faithful propagation of the human neoplastic cells. We showed that the histological architecture of the original tumors is maintained in the PDX models for different grades of differentiation, even after extensive passages. Moreover, although the non-malignant stroma of the human tumor is replaced with cells from the host mouse, these components seem to mimic several features of the original tumor stroma, including cancer-associated fibroblasts (CAFs) and tumor vasculature [26–28]. In PDAC, the CAFs are largely responsible for the production of the extracellular matrix found in the tumor stroma, and the production of collagen by CAFs is mediated by their close association with PDAC cells [29,30]. Despite the replacement of human CAFs by murine fibroblasts, we demonstrated that the collagen structures found in PDX models of PDAC closely resemble those found in the original tumor. Therefore, PDX tumors provide a system to investigate the cellular and non-cellular components of the stroma and their interactions with PDAC cells.

The pathological factors associated with PDX formation have been described for a variety of cancers [31,32]. However, their identification in PDAC has largely remained elusive, with a single study correlating tumor size (> 3.5 cm) with successful xenograft generation [16,18,19]. In our series, we identified lymphovascular invasion and lymph node metastasis as potential determinants for PDX formation and rapid growth, respectively. An important role for lymphovascular invasion is highlighted by the fact that only 14% of tumors without lymphovascular invasion successfully engrafted. These results suggest that engraftment may reflect an underlying biological mechanism that allows these tumors to adapt and grow in a new environment. Interestingly, previous studies employing NOD/SCID mice have failed to make similar correlations with these pathological features and PDAC engraftment and growth [16–18]. While the more immunocompromised background of these mice likely allows for more efficient tumor engraftment, it is possible that the reduced selective pressure of NOD/SCID mice masks the inherent biological differences of patient tumors.

The association of molecular alterations with PDAC PDX formation has been controversial. The focus of this analysis has been on the loss of SMAD4 expression, which has been implicated in metastatic spread of PDAC [33]. Previously, Garrido-Laguna et al. found that engrafted tumors were more frequently associated with SMAD4 inactivation [17]. In contrast, in a study by Jun et al. there was no association between loss of SMAD4 and success of tumor engraftment [16]. Similarly, our analysis of SMAD4 expression in patient tumors did not find any correlation with the success or timing of tumor engraftment. Moreover, our study and others have demonstrated that successfully and unsuccessfully engrafted tumors exhibit a similar distribution of *KRAS* mutations and comparable expression or mutation of *TP53*. Collectively, these results suggest that that single genetic alterations may not be determinant of tumor engraftment, allowing PDX models of PDAC to recapitulate the broad genetic diversity of patient tumors.

As seen in PDX models of other cancers, successful engraftment of PDAC tumors correlates with shorter recurrence-free and overall survival. In the present study, tumors successfully engrafted in mice showed significantly poorer overall survival and xenograft formation was an independent predictor of poor survival. Similarly, Garrido-Laguna et al. showed that patients whose pancreatic tumors failed to engraft had an 81% reduced risk of death, and Thomas et al. demonstrated that patients whose tumors successfully engrafted experienced recurrence significantly earlier than those whose did not grow [17,18]. Despite the shorter survival of patients with PDAC that form PDX tumors, most tumors are established in mice 4–5 months before recurrence in patients undergoing surgical resection. In our cohort of patients with recurrent disease, xenograft tumors developed a median of 166 days before diagnosis of recurrence. Similarly, Thomas et al. detected first palpable signs of tumor formation a median of 134.5 days before radiographic recurrence identification [18]. Given the limited number of therapeutic options and the variable rate of successful growth, the systematic use of PDX tumors for real-time chemo-sensitivity testing is not practical at this time. However, by predicting recurrence months before current surveillance modalities, these models might provide a window of opportunity for increased surveillance and differentiation of treatment, especially in patients at higher risk of recurrent disease.

Our study had limitations. The patient population derives from a single referral center, which may result in variations relative to the general population. For instance, nearly a third of patients underwent neoadjuvant treatment. However, this was not associated with the rate of tumor engraftment; moreover, multivariable-adjusted survival models adjusted for different peri-operative treatment status, minimizing any potential biases. A small proportion (16%) of patients in our study had metastatic disease at the time of surgery, introducing heterogeneity in analyses of OS. To address this issue, we conducted a separate analysis of RFS among the more homogeneous subset of patients with resectable primary tumors and no evidence of metastatic disease.

In conclusion, successful establishment of PDAC PDX predicts an increased risk of disease recurrence and mortality in the original patients. Lymphovascular invasion and lymph node positivity might reflect an underlying biological mechanism that allows these tumors to establish and thrive in a new host environment. These models are able to faithfully reproduce the cancer and stromal architecture from the original tumor, and may therefore be valuable tools to test new therapeutic alternatives and identify patients who are at very high risk of disease recurrence following resection.
